# Erratum to “Establishment of a Consistent L929 Bioassay System for TNF-*α* Quantitation to Evaluate the Effect of Lipopolysaccharide, Phytomitogens and Cytodifferentiation Agents on Cytotoxicity of TNF-*α* Secreted by Adherent Human Mononuclear Cells”

**DOI:** 10.1155/2017/7327215

**Published:** 2017-03-16

**Authors:** Ming-Yuh Shiau, Hui-Ling Chiou, Yao-Ling Lee, Tzer-Min Kuo, Yih-Hsin Chang

**Affiliations:** ^1^Hung Kuang Institute of Technology, Chung Shan Medical University, Taichung 402, Taiwan; ^2^School of Medical Technology, Chung Shan Medical University, Taichung 402, Taiwan; ^3^Institute of Immunology, Chung Shan Medical University, Taichung 402, Taiwan

In the article titled “Establishment of a Consistent L929 Bioassay System for TNF-*α* Quantitation to Evaluate the Effect of Lipopolysaccharide, Phytomitogens, and Cytodifferentiation Agents on Cytotoxicity of TNF-*α* Secreted by Adherent Human Mononuclear Cells” [1], the figures were missing due to a publisher error. The figures are as follows.

## Figures and Tables

**Figure 1:**
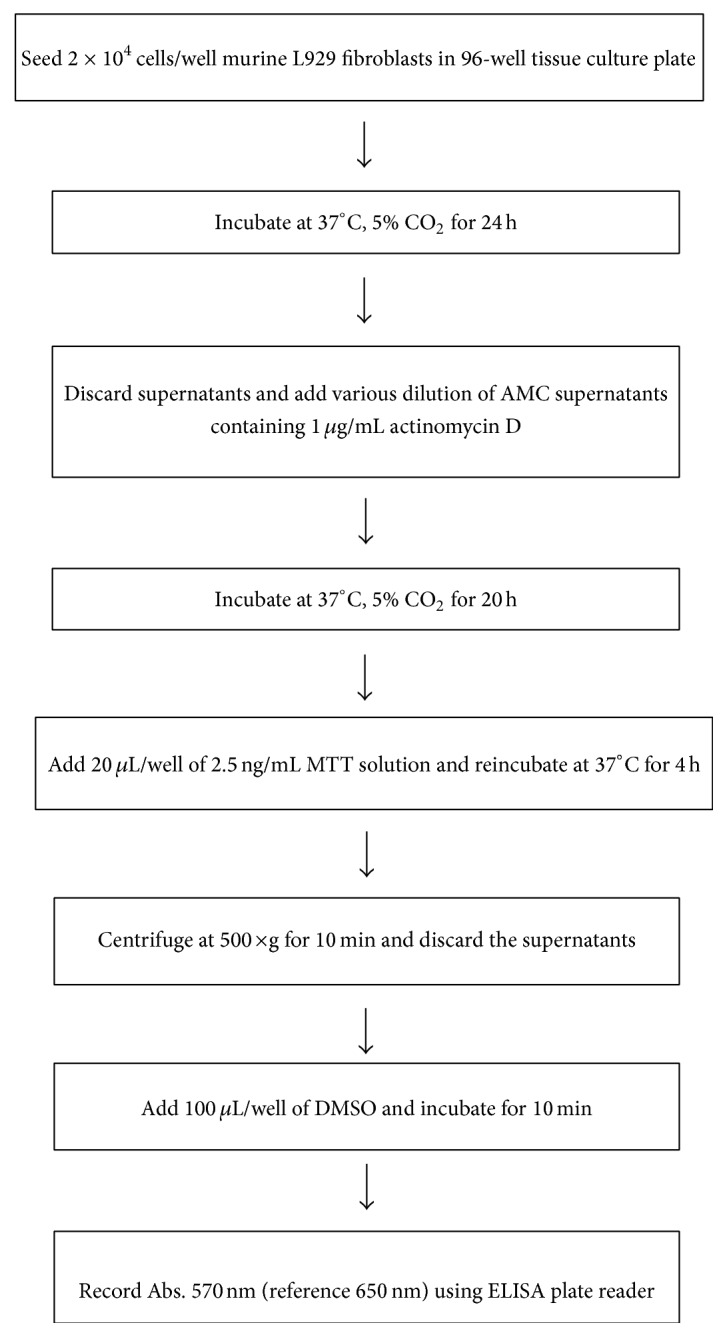
Flowchart of modified L929 cytotoxic bioassay system for TNF-*α* quantitation.

**Figure 2:**
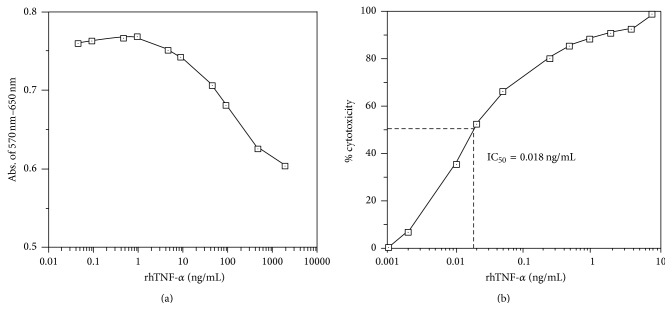
(a) Dose-response curve of human HEp-2 cells in response to rhTNF-*α*. Various concentrations of rhTNF-*α* were diluted with complete RPMI-1640 medium. Details were described in Materials and Methods. Positive control wells containing 200 ng/mL rhTNF-*α* were used as blank. IC_50_ of rhTNF-*α* to HEp-2 cells was estimated to be more than 10 ng/mL. (b) Dose-response curve of murine L929 fibroblasts in response to rhTNF-*α*. Details of L929 bioassay and MTT stain were described in Materials and Methods. IC_50_ of rhTNF-*α* to L929 cells was estimated to be 0.18 ng/mL.

**Figure 3:**
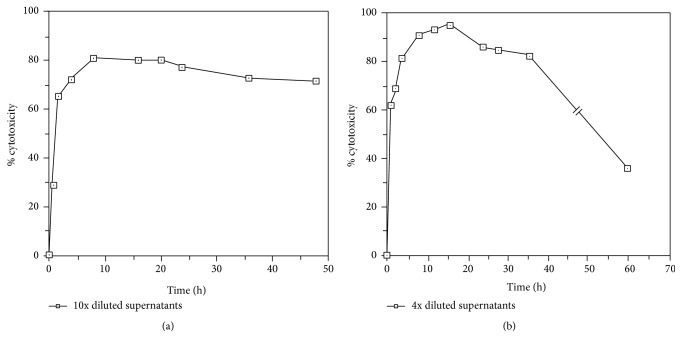
(a) Time course of TNF cytotoxicity produced by AMC cultured in complete RPMI-1640 containing 0.02 *μ*g/mL LPS. Crude supernatants were harvested at time indicated and TNF cytotoxicity in 10x diluted cell-free supernatants was analyzed. (b) Time course of TNF cytotoxicity produced by AMC activated under 1 h of 0.02 *μ*g/mL LPS stimulation. Crude supernatants were harvested at time indicated and TNF cytotoxicity in 4x diluted cell-free supernatants was assayed.

**Figure 4:**
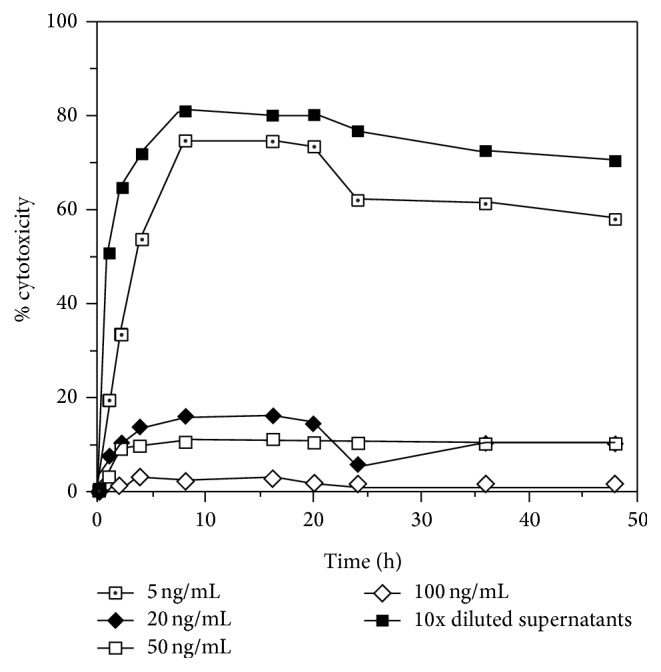
Neutralization curve of TNF-*α* cytotoxicity in LPS-stimulated AMC supernatants by anti-TNF-*α* monoclonal antibody. TNF-*α* cytotoxicity in 10x diluted AMC supernatants containing final concentration of 5, 20, 50, or 100 ng/mL anti-TNF-*α* monoclonal antibody was assayed. Details were described in Materials and Methods.

**Figure 5:**
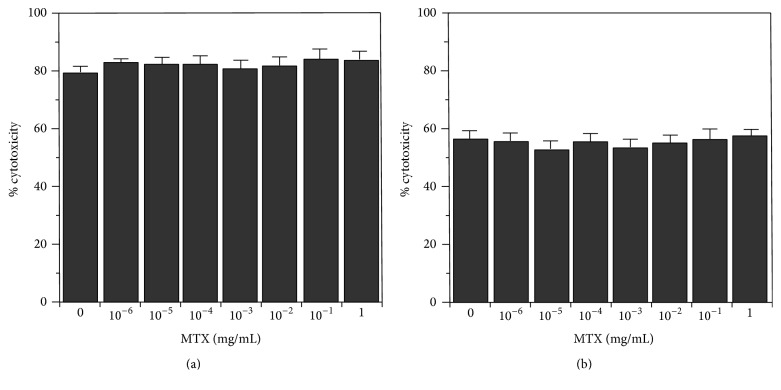
Effect of MTX on TNF-*α* cytotoxicity. AMC were activated by 0.02 *μ*g/mL LPS in the presence of MTX at various concentrations (1 ng/mL–1 mg/mL). After overnight culture, cell was washed to remove the LPS and MTX and then subsequently further incubated in the presence (a) or absence (b) of MTX for 24 h to allow TNF-*α* secretion. TNF cytotoxicity in 10x (a) or 5x (b) diluted supernatants was analyzed.

**Figure 6:**
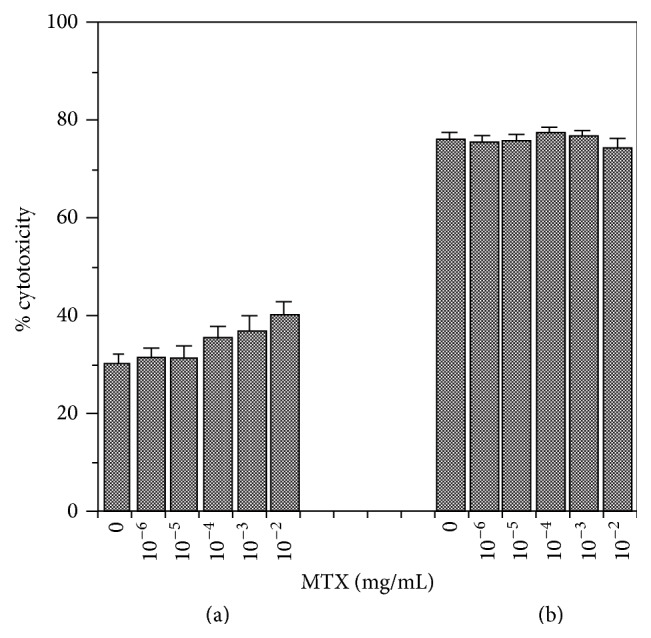
Effect of MTX on cytotoxicity of TNF-*α* secreted by LPS-pulsed AMC. After 1 h of 0.02 *μ*g/mL LPS stimulation, AMC were washed and further incubated for 2 h (a) or overnight (b) in the presence of MTX at various concentration (10^−6^ to 10^−2^ mg/mL). TNF cytotoxicity in 10x diluted supernatants was analyzed.

**Figure 7:**
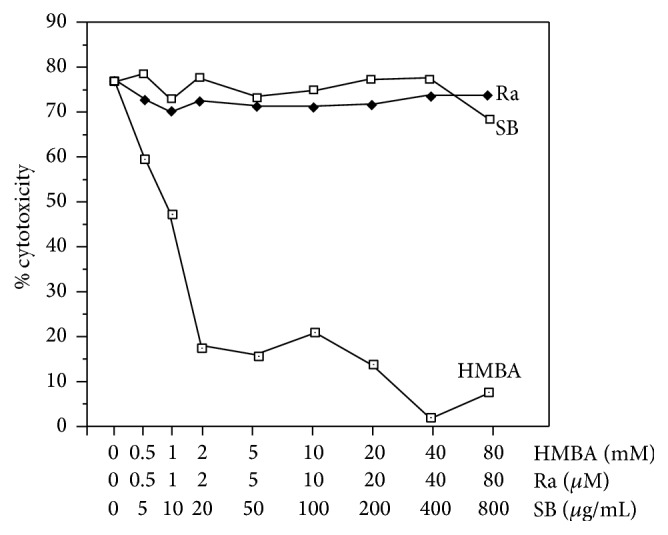
Effect of cytodifferentiation agents (HMBA, Ra, and SB) on cytotoxicity of TNF-*α* secreted by AMC. AMC were activated with 0.02 *μ*g/mL LPS in the presence of cytodifferentiation agents at various concentrations (as summarized in Table  1). After overnight culture, cell-free supernatants were harvested and TNF cytotoxicity in 10x diluted supernatants was analyzed.

**Figure 8:**
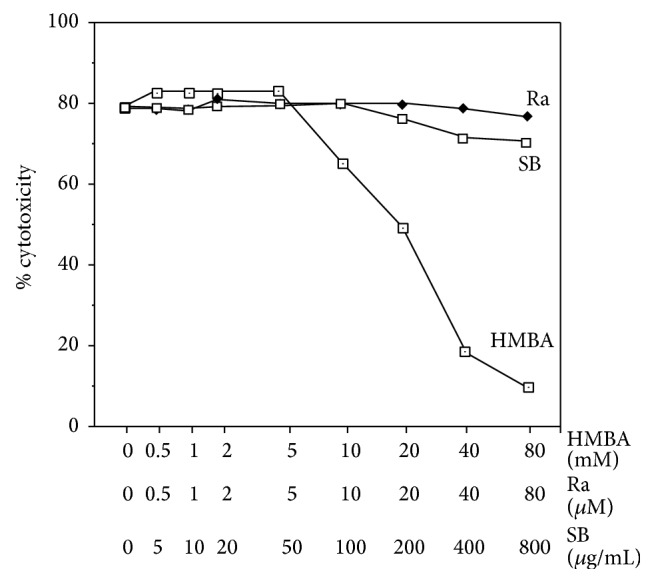
Effect of cytodifferentiation agents on cytotoxicity of TNF-*α* secreted by LPS-pulsed AMC. After 1 h of 0.02 *μ*g/mL LPS stimulation, AMC were washed and further incubated overnight in the presence of cytodifferentiation agents at various concentrations (as summarized in Table  1). TNF cytotoxicity in 5x diluted supernatants was analyzed.

## References

[1] Shiau M.-Y., Chiou H.-L., Lee Y.-L., Kuo T.-M., Chang Y.-H. (2001). Establishment of a consistent L929 bioassay system for TNF-*α* quantitation to evaluate the effect of lipopolysaccharide, phytomitogens and cytodifferentiation agents on cytotoxicity of TNF-*α* secreted by adherent human mononuclear cells. *Mediators of Inflammation*.

